# A bibliometric review about scientific trends and advances on residual feed intake (RFI) and feed efficiency in ruminants

**DOI:** 10.1007/s11250-026-05050-5

**Published:** 2026-05-08

**Authors:** Josiel Ferreira, Lucas Ferreira Gonçalves, Tiago do Prado Paim, Sarah Figueiredo Martins Bonilha, Joslaine Noely dos Santos Goncalves Cyrillo, Maria Eugênia Zerlotti Mercadante, Renata Helena Branco Arnandes, Ricardo Lopes Dias da Costa, Concepta McManus

**Affiliations:** 1 Instituto Federal de Educação, Ciência e Tecnologia Goiano, Campus Campos Belos, GO-118 Highway, Qd. 1-A, Lt. 1, Novo Horizonte District, Campos Belos, GO 73840-000 Brazil; 2 Instituto Federal de Educação, Ciência e Tecnologia Goiano, Campus Iporá, GO 75200-000 Brazil; 3 Departamento de Pesca e Aquicultura, Empresa Brasileira de Pesquisa Agropecuária (EMBRAPA), Palmas, TO 77008-900 Brazil; 4 Centro de Pesquisa Pecuária Sustentável, Instituto de Zootecnia, Sertãozinho, SP 14174-000 Brazil; 5 Centro de Pesquisa Pecuária Sustentável, Instituto de Zootecnia, São José do Rio Preto, SP 15130-000 Brazil; 6 Centro de Pesquisa e Desenvolvimento de Zootecnia Diversificada, Instituto de Zootecnia, Nova Odessa, SP 1338-0011 Brazil; 7 Universidade de Brasília (UnB), Campus Darcy Ribeiro, Brasília, DF 70910-900 Brazil; 8 Centro de Energia Nuclear na Agricultura (CENA), Universidade de São Paulo (USP), Piracicaba, SP 13416-000 Brazil

**Keywords:** Cattle, Genetic improvement, Methane emission, Sheep, Sustainability

## Abstract

The increasing demand for sustainable livestock production systems has intensified scientific interest in feed efficiency traits, particularly residual feed intake (RFI), as a strategy to improve productivity while reducing environmental impacts. This study provides a comprehensive bibliometric assessment of global scientific production on RFI and feed efficiency in ruminants. A total of 2632 documents indexed in Scopus and Web of Science databases between 2015 and 2024 were analyzed, covering 374 scientific sources and involving 8,406 authors. Scientific output showed a strong and consistent upward trend throughout the study period, with an annual growth rate of 48.9%, particularly from 2019 onwards. Brazil, United States, Australia, Canada, and China were identified as the leading contributors to research development in this field. Authorship patterns revealed a highly collaborative research structure, with an average of 6.97 co-authors per publication, reflecting the interdisciplinary nature of feed efficiency studies. Keyword co-occurrence and network analyses highlighted the close integration of RFI research with themes related to sustainability, methane emissions, genetics, nutrition, and rumen biology. Overall, the results indicate that research on RFI has evolved from a predominantly zootechnical indicator toward a consolidated and sustainability-oriented scientific domain, reinforcing its strategic importance for the development of climate-smart ruminant production systems.

## Introduction

The growing global demand for animal-derived products has intensified the search for livestock production systems that are more efficient, sustainable, and economically viable. The concept of residual feed intake (RFI), first proposed by Koch et al. (1963), has become one of the most widely used indicators of feed efficiency in ruminants, allowing the identification of animals that consume less feed than expected for a given level of production and body development without compromising performance. Improvements in feed efficiency are directly associated with reduced production costs and lower environmental impacts, particularly through reductions in enteric methane emissions (Knapp et al. 2014; Goldansaz et al. 2017; Martin et al. 2021; Ferreira et al. 2024; da Silva Soares et al. 2025).

Over recent decades, advances in phenotyping, genotyping, and statistical approaches have contributed to a deeper understanding of the biological mechanisms underlying variation in RFI, enabling its application across diverse breeds, environments, and production systems (Pires et al. 2021; Parsons et al. 2021; Rodrigues et al. 2024; Matos et al. 2024; Ferreira et al. 2025). More recently, the scientific relevance of feed efficiency has expanded beyond its traditional role as a nutritional and zootechnical indicator. Increasing attention has been given to its relationship with sustainability challenges, including methane mitigation, resource-use efficiency, and climate resilience in livestock systems (Martin et al. 2021). This evolution reflects a broader paradigm shift in animal science, in which production efficiency is increasingly integrated with environmental and societal demands.

The importance of feed efficiency is particularly evident in tropical and subtropical production systems, where livestock are frequently exposed to environmental constraints such as heat stress, seasonal variability in forage availability and quality, and parasite challenges. In these contexts, improving feed efficiency through traits such as RFI may contribute to enhancing productivity, resilience, and sustainability in systems based on locally adapted breeds and extensive management conditions (Pires et al. 2021; Rodrigues et al. 2024). Consequently, RFI research has gained strategic relevance for the development of climate-smart livestock production systems in tropical regions.

Despite its increasing scientific and practical relevance, research on RFI and feed efficiency has expanded rapidly and now encompasses multiple disciplinary perspectives, including genetics, nutrition, physiology, microbiology, and environmental sciences. This rapid expansion and conceptual transition highlight the need for an updated and comprehensive assessment of global scientific trends in RFI research. In this context, bibliometric analysis represents a valuable approach to systematically map scientific production, identify knowledge gaps, and evaluate the evolution of research activity over time (Marino et al. 2023; McManus et al. 2023).

Therefore, the aim of this study was to conduct a bibliometric analysis of global scientific production on residual feed intake and feed efficiency in ruminants, focusing on publications indexed between 2015 and 2024. Specifically, this study sought to characterize publication trends, leading countries, institutions, authors, collaboration patterns, and the main thematic areas shaping the scientific development of feed efficiency research. Although the bibliometric dataset was restricted to this period to ensure consistency in database indexing, selected studies published in 2025 were cited to provide updated scientific context.

## Materials and methods

### Data sources, processing, and eligibility criteria

Bibliographic data used in this study were retrieved from Scopus and Web of Science (WoS) databases through the exportation of complete records in BibTeX format. After importation into R statistical environment, records from both databases were integrated into a single dataset using the bibliometrix package, with automatic identification and removal of duplicate documents across sources.

A multi-step filtering procedure was subsequently applied to ensure the thematic coherence and relevance of the final bibliographic dataset. Initially, a negative document-level filter was performed based on the analysis of titles, abstracts, and keywords. In this step, only publications clearly outside the scope of the study were excluded, including studies involving non-ruminant species (e.g., swine, poultry, and fish), human-related research, and documents belonging to research areas unrelated to the central topic of feed efficiency. In addition, publications indexed for the year 2025 were removed in order to avoid the inclusion of records still undergoing database indexing. Nevertheless, a limited number of recent studies published in 2025 were considered in the narrative sections of the manuscript to ensure an updated scientific background.

Subsequently, a positive thematic filter was applied to ensure the relevance of the retained documents. This filter consisted of keeping only articles that contained, in at least one of the textual fields, terms associated with ruminants, feed intake, or feed efficiency. These included expressions related to dry matter intake, energy efficiency, residual feed intake, and ruminal processes. This strategy allowed the exclusion of remaining non-related documents that had not been removed in the previous step, such as studies from biomedical sciences, without compromising the coverage of relevant literature.

After defining the final document dataset, bibliometric analyses were conducted using the functionalities of bibliometrix and its graphical interface biblioshiny. For semantic analyses, including word frequency analysis, word clouds, and conceptual structure mapping, an additional text-cleaning procedure was applied. This step was restricted to the removal of generic terms and descriptors not directly related to feed efficiency mechanisms or indicators and was applied exclusively to textual analyses, without any further exclusion of documents from the dataset.

This multi-step procedure resulted in a thematically consistent and semantically refined bibliographic database, allowing a more accurate representation of scientific production related to feed efficiency and residual feed intake in ruminants.

**Fig. 1:**
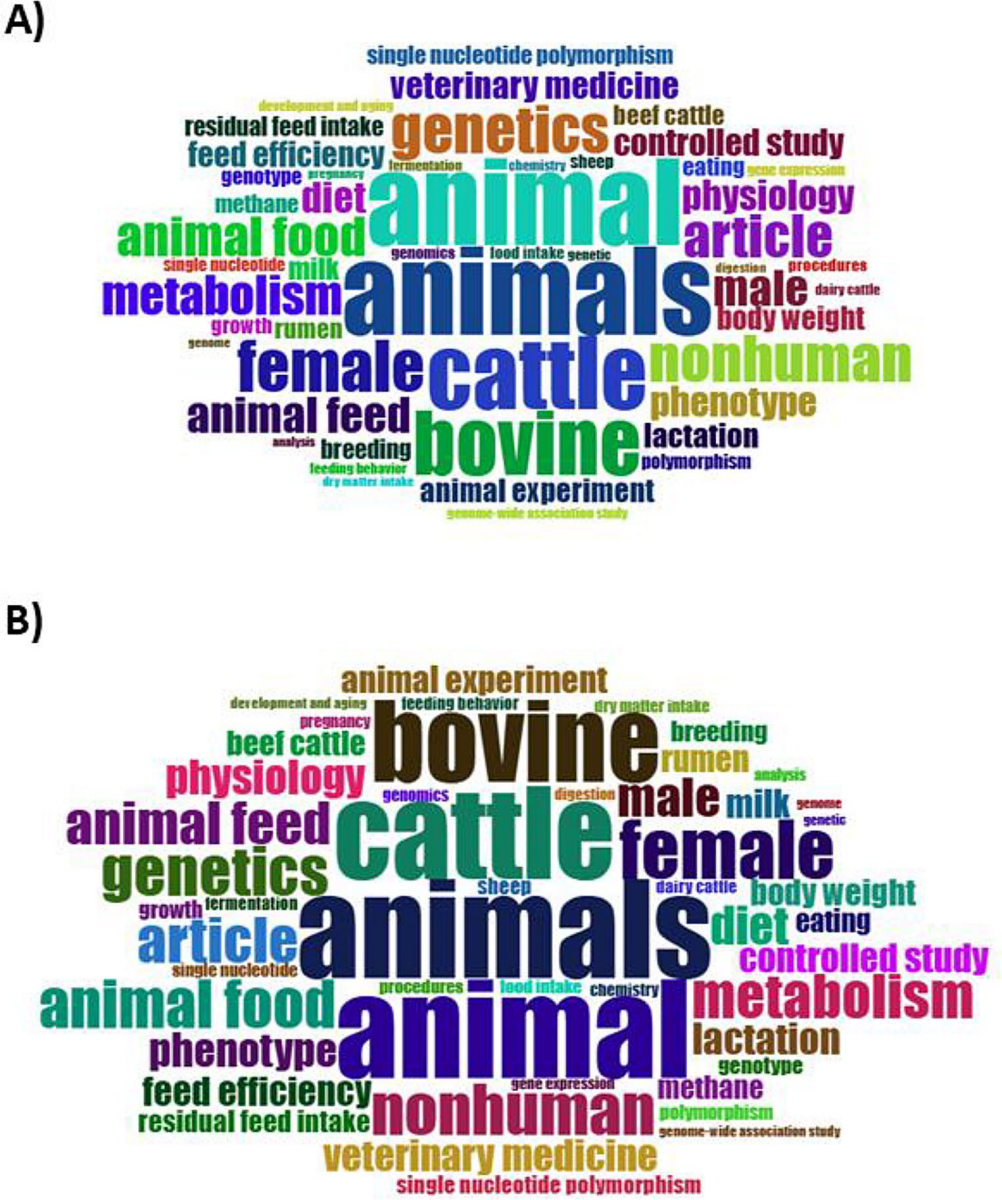
Word clouds of keywords related to residual feed intake and feed efficiency in ruminants before and after the text-cleaning procedure applied for semantic analyses. (**A**) Word cloud generated from the original dataset prior to keyword cleaning. (**B**) Word cloud generated after the removal of generic terms and non-specific descriptors, applied exclusively to textual analyses, without additional exclusion of documents

For semantic analyses, including word frequency analysis, word clouds, and conceptual structure mapping, an additional text-cleaning procedure was applied. This step was restricted to the removal of generic terms and descriptors not directly related to feed efficiency mechanisms or indicators and was applied exclusively to textual analyses, without any further exclusion of documents from the dataset. The impact of this cleaning procedure on keyword representation is illustrated in Fig. 1.

### Bibliometric analysis

The bibliometric analyses were conducted using the bibliometrix package in R v.4.2.3? (Core Team 2021) and its graphical interface biblioshiny (Aria and Cuccurullo 2017), which were used to compute descriptive and relational bibliometric indicators. The analyses included: (i) annual scientific production and document types; (ii) most productive and influential journals, authors, institutions, and countries; and (iii) collaboration patterns among authors, institutions, and countries.

Network analyses were performed to explore relationships among countries, institutions, authors, and keywords, based on co-authorship and co-occurrence matrices. Keyword co-occurrence analyses were used to identify the conceptual structure of the field and to highlight major research themes and emerging topics related to feed efficiency and residual feed intake in ruminants.

For visualization and network mapping, biblioshiny outputs were complemented using VOSviewer (version 1.6.18; van Eck and Waltman 2010), enabling clearer representation of collaboration networks and keyword relationships. Default normalization and clustering algorithms implemented in the software were applied.

This analytical framework allowed the identification of publication trends, knowledge structures, and collaboration patterns, providing a comprehensive overview of the scientific development of feed efficiency and residual feed intake research in ruminants.

## Results

### General bibliometric characteristics

The bibliometric analysis comprised a total of 2632 documents published between 2015 and 2024, after integrating records retrieved from Scopus and Web of Science and applying the eligibility and thematic filtering procedures described in Sect.  2.1 (Table 1). Prior to duplicate removal and thematic refinement, the initial dataset contained 3174 records, indicating a substantial reduction aimed at improving thematic coherence.

**Table 1:** Bibliometric parameters for publications on Residual Feed Intake and Feed Efficiency in ruminants of publications indexed in Scopus

Description	Results
Main information about data	
Timespan	2015:2024
Sources (Journals, Books, etc.)	374
Documents	2,632
Annual growth rate %	48.88
Document average age	4.6
Average citations per doc	15.16
Document contents	
Keywords plus	7,387
Author’s keywords	4,862
Authors	
Authors	8,406
Authors of single-authored docs	43
Authors collaboration	
Single-authored documents	49
Co-Authors per documents	6.97

The final dataset encompassed publications distributed across 374 sources, authored by 8,406 researchers, reflecting the broad disciplinary scope and international nature of research on RFI and feed efficiency in ruminants. The mean number of citations per document was 15.16, suggesting a combination of good citation accumulation considering the recent publication of these papers. Authorship patterns revealed a predominance of collaborative research, with an average of 6.97 co-authors per document, while single-authored publications represented a small proportion of the dataset (Table 1).

### Temporal evolution of scientific production

**Fig. 2:**
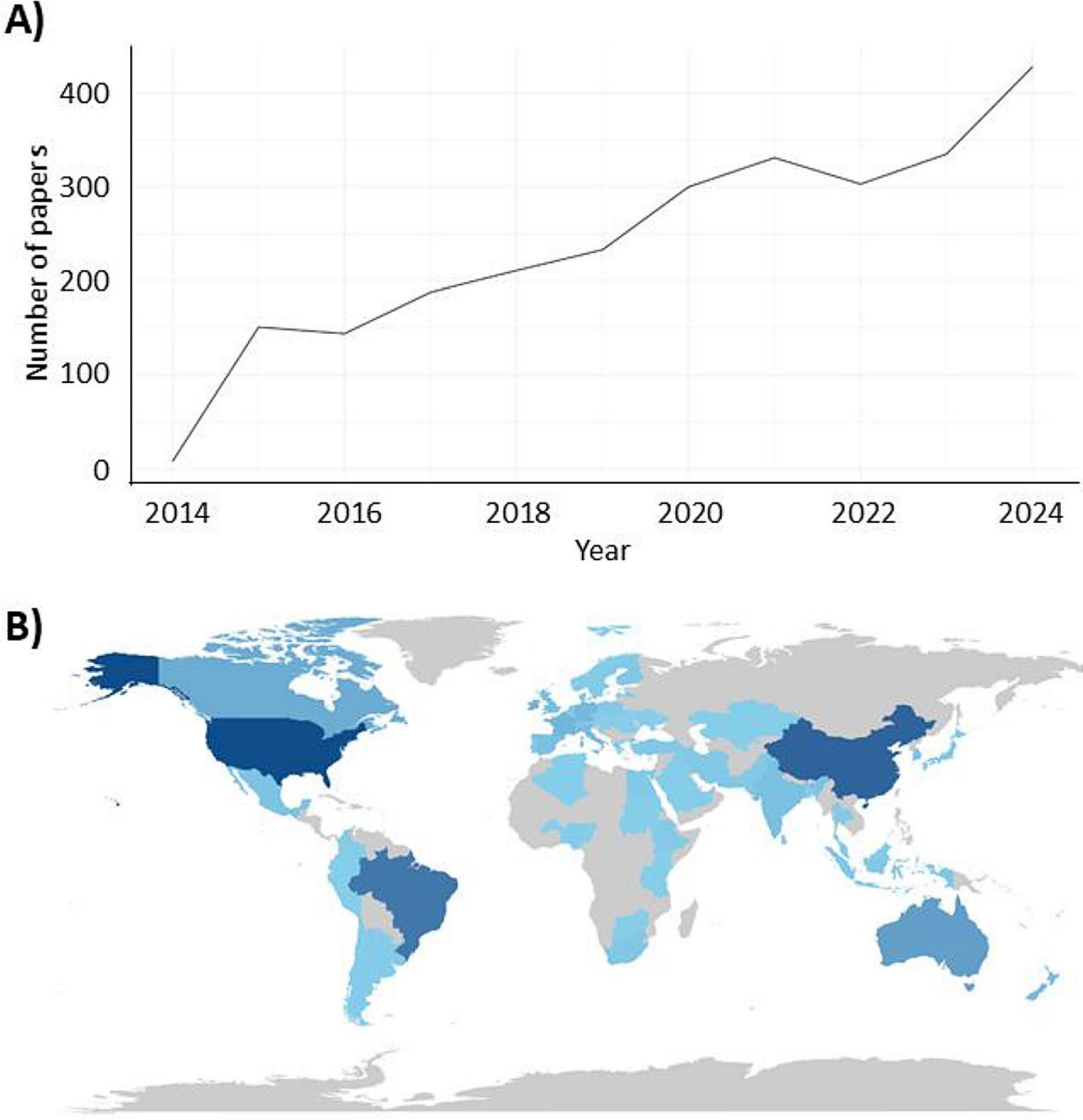
Geographic distribution and temporal evolution of scientific production on residual feed intake and feed efficiency in ruminants. (**A**) Global map showing the number of publications by country based on author affiliations. (**B**) Annual scientific production of the most productive countries between 2014 and 2024

The annual scientific production related to residual feed intake and feed efficiency in ruminants exhibited a consistent upward trend throughout the analyzed period (Fig. 2). From 2015 to 2018, publication output increased gradually followed by a more pronounced growth from 2019 onward. The highest publication volumes were observed in most recent years of the time series. This temporal pattern indicates a progressive expansion of scientific activity in the field, with increasing numbers of studies addressing feed efficiency and RFI in different ruminant species and production contexts.

### Geographic distribution of scientific production

Scientific production on RFI and feed efficiency was geographically widespread, with contributions from countries across all major livestock-producing regions (Fig. 2A). Brazil presented the highest number of publications, followed by the United States, Australia, Canada, and China.

The annual scientific production of the most productive countries showed distinct temporal patterns (Fig. 2B). Brazil and the United States maintained consistently high publication output throughout the study period, while Australia, Canada, and China exhibited a gradual increase in scientific production, particularly in the latter half of the analyzed timeframe.

**Fig. 3:**
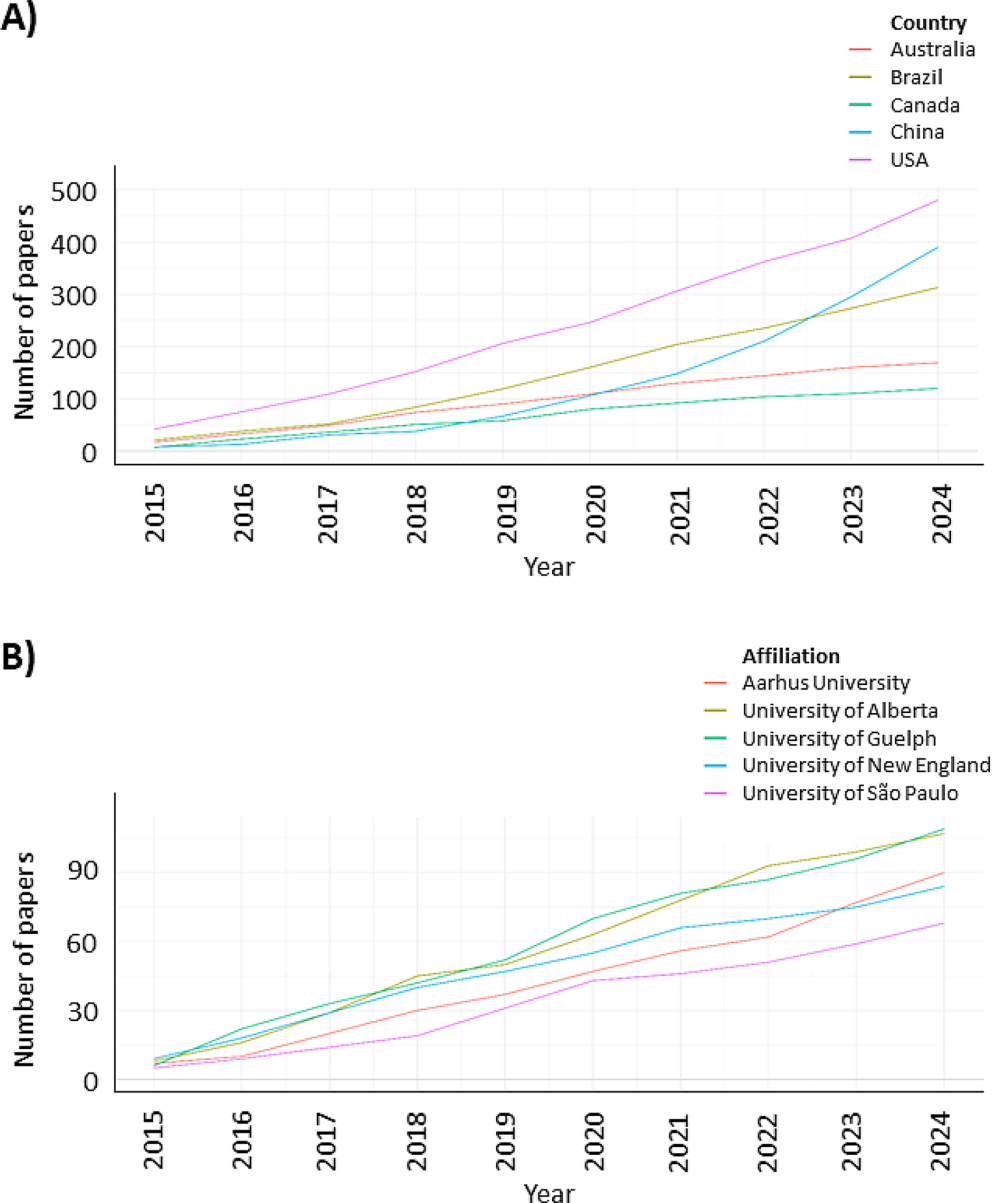
Temporal trends in scientific production on residual feed intake and feed efficiency in ruminants. (**A**) Annual number of publications for the five most productive countries. (**B**) Annual number of publications for the five most productive affiliations over the study period

At the institutional level, a similar temporal pattern was observed among the most productive affiliations, with sustained or increasing publication output over time (Fig. 3B), indicating the presence of established research centers actively contributing to the advancement of feed efficiency research.

### Source, scientific leadership, and collaboration networks

**Fig. 4:**
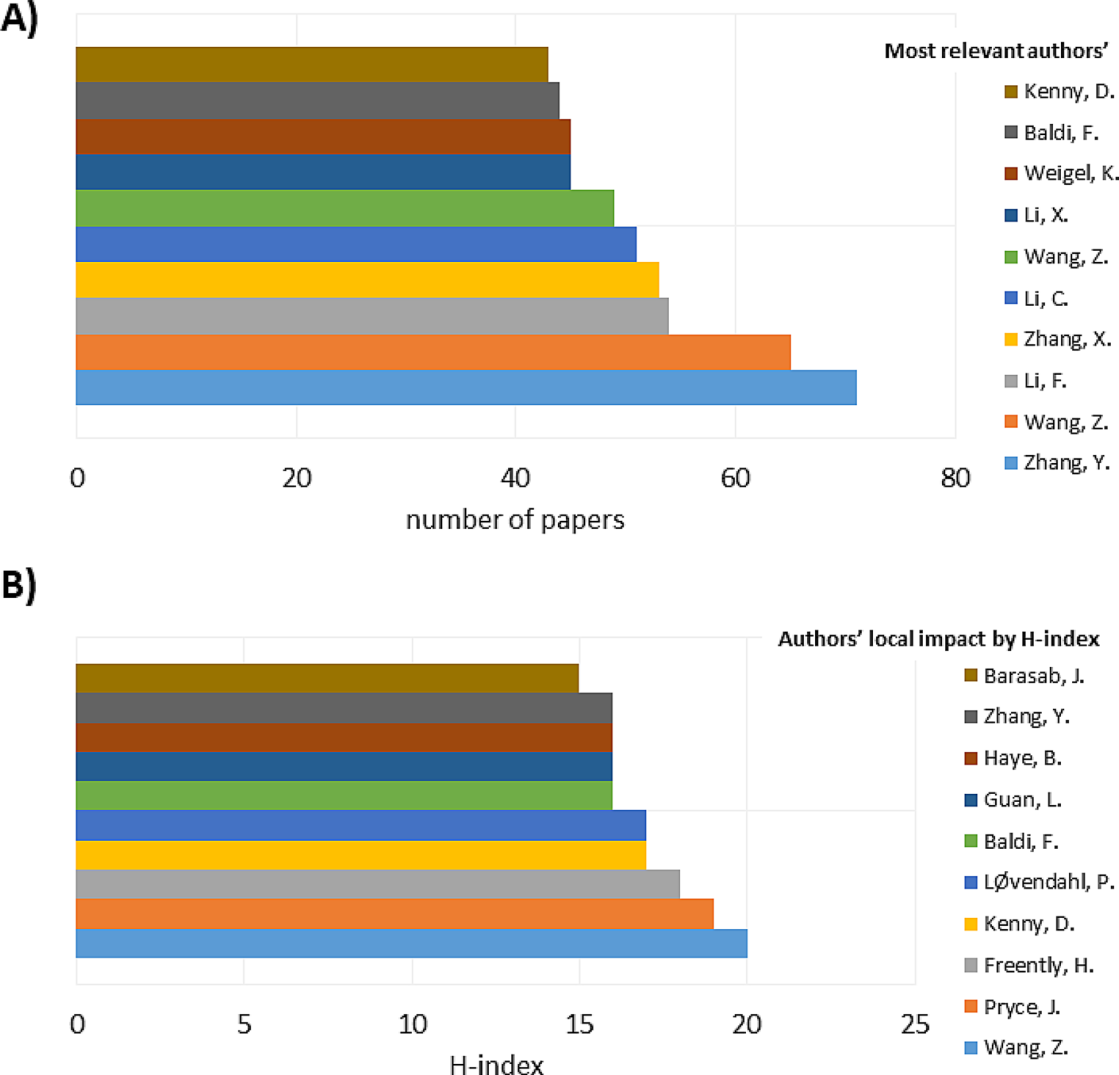
Scientific productivity and impact of leading authors in research on residual feed intake and feed efficiency in ruminants. (**A**) Total number of publications by the most productive authors. (**B**) H-index values of the most productive authors based on the analyzed dataset

The most cited publications identified in the dataset were primarily related to themes such as rumen microbiome, methane emissions, and biological determinants of feed efficiency (Table 2). Author-level analysis indicated a concentration of scientific output among a limited group of highly productive researchers, alongside a larger number of authors with lower individual publication counts (Fig. 4A). The H-index values calculated from the analyzed dataset further highlighted differences in scientific impact among the most productive authors (Fig. 4B).

**Table 2:** Top 10 most cited papers in research on residual feed intake and feed efficiency in ruminants

Reference	Title	Source title	Cited by	Doi
Myer et al. (2015)	Rumen microbiome from steers differing in feed efficiency	PLOS One	312	10.1371/journal.pone.0129174
Wallace et al. (2015)	The rumen microbial metagenome associated with high methane production in cattle	BMC Genomics	289	10.1186/s12864-015-2032-0
Tapio et al. (2017)	The ruminal microbiome associated with methane emissions from ruminant livestock	Journal of Animal Science and Biotechnology	281	10.1186/s40104-017-0141-0
Li and Guan (2017)	Metatranscriptomic profiling reveals linkages between the active rumen microbiome and feed efficiency in beef cattle	Applied and Environmental Microbiology	265	10.1128/AEM.00061-17
Jewell et al. (2015)	Ruminal bacterial community composition in dairy cows is dynamic over the course of two lactations and correlates with feed efficiency	Applied and Environmental Microbiology	224	10.1128/AEM.00720-15
Cantalapiedra-Hijar et al. (2018)	Review: Biological determinants of between-animal variation in feed efficiency of growing beef cattle	Animal	176	10.1017/S1751731118001489
D’Occhio et al. (2019)	Influence of nutrition, body condition, and metabolic status on reproduction in female beef cattle: A review	Theriogenology	174	10.1016/j.theriogenology.2018.11.010
Kenny et al. (2018)	Invited review: Improving feed efficiency of beef cattle – the current state of the art and future challenges	Animal	170	
O’Hara et al. (2020)	The role of the gut microbiome in cattle production and health: driver or passenger?	Annual Review Animal Biosciences	168	
Charmley et al. (2016)	A universal equation to predict methane production of forage-fed cattle in Australia	Animal Production Science	165	

**Fig. 5:**
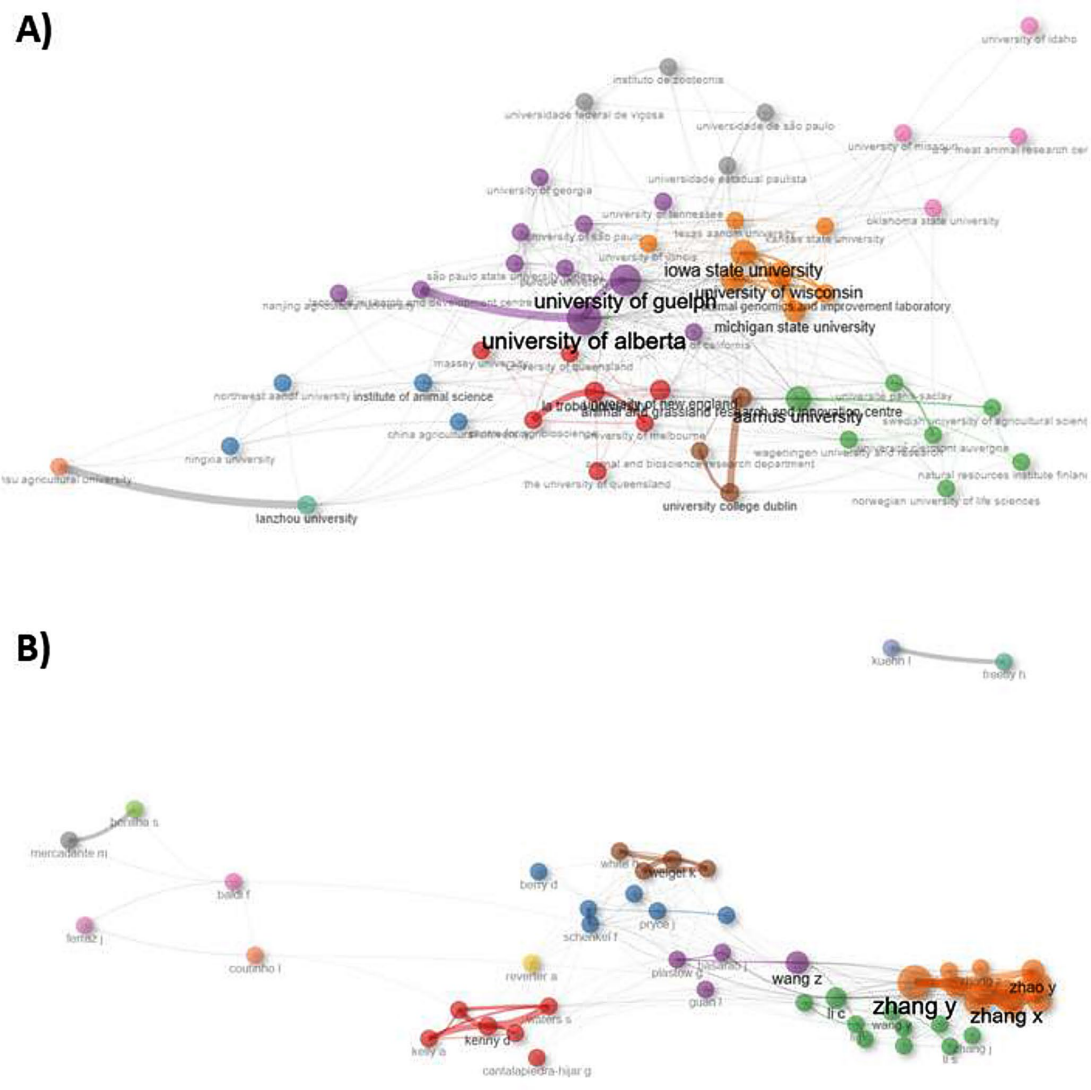
Collaboration patterns in research on residual feed intake and feed efficiency in ruminants. (**A**) International collaboration network among countries based on co-authorship relationships. (**B**) Strength of collaborative links among the most connected countries, derived from co-authorship analysis

Collaboration network analysis based on co-authorship relationships demonstrated extensive international collaboration among countries engaged in feed efficiency research (Fig. 5A). Strong collaborative links were observed among the most connected countries, indicating the formation of international research networks focused on residual feed intake and related topics (Fig. 5B). Overall, these results illustrate a research field characterized by high levels of collaboration, stable scientific leadership, and increasing international integration.

## Discussion

The present bibliometric analysis provides a structured overview of the scientific development of research on residual feed intake (RFI) and feed efficiency in ruminants between 2015 and 2024. By integrating publication trends, geographic distribution, source structure, authorship patterns, and collaboration networks, the results depict a research field that has expanded rapidly while simultaneously consolidating its conceptual and institutional foundations.

### Expansion and consolidation of feed efficiency research

The sustained increase in scientific production observed over the analyzed period reflects the growing recognition of feed efficiency as a strategic trait in ruminant production systems. This trend can be interpreted as evidence that RFI research has transitioned from a specialized topic toward a central component of animal science research agendas.

Over the last decade, RFI research has increasingly incorporated integrative approaches that combine nutrition, host genetics, rumen microbiome dynamics (Liu et al. 2022; Lima et al. 2024), metabolic efficiency (Xie et al. 2022), and environmental responses (Matos et al. 2024). This shift reflects a transition from viewing RFI as a simple indicator of feed intake deviation toward recognizing it as a complex phenotype associated with energy partitioning, physiological adaptability, and resilience under variable production conditions. This expansion should not be viewed solely as an increase in publication output, but also as an indicator of important conceptual changes in the biological understanding of feed efficiency. Similar publication trajectories have been reported in bibliometric studies addressing methane mitigation, climate resilience, and precision livestock farming, suggesting that feed efficiency research has become embedded within a wider sustainability-oriented framework rather than remaining an isolated zootechnical metric.

In particular, the growing scientific attention to methane emissions, digestive efficiency, and animal robustness indicates that RFI is increasingly being investigated as a proxy for biological efficiency and environmental performance (Velazco et al. 2017; Batalha et al. 2020; Sakamoto et al. 2021). Consequently, the observed growth in scientific production also reflects the emergence of new research paradigms in which productivity, sustainability, and adaptation to environmental stressors are considered simultaneously.

### Geographic leadership and global research dynamics

The geographic distribution of scientific output reflects the strategic importance of feed efficiency research in countries with large ruminant production sectors. Long-term investments in genetic improvement, nutrition, and sustainability-oriented livestock research have contributed to the leadership observed for Brazil, United States, Australia, Canada, and China. Similar geographic patterns have been reported in global reviews of feed efficiency research, which highlight the role of structured breeding programs and technological adoption in driving scientific productivity (Kenny et al. 2018; Martin et al. 2021).

Beyond differences in publication volume, the geographic distribution of studies reflects the diversity of production systems in which residual feed intake (RFI) is investigated. Feed efficiency has been evaluated under contrasting climatic conditions and management strategies, ranging from intensive confinement systems to extensive grazing environments. These differences are particularly relevant because environmental factors influence physiological responses, metabolic efficiency, and animal performance (Pires et al. 2021; Matos et al. 2024).

In tropical and subtropical regions, where livestock production is frequently exposed to heat stress, seasonal forage variability, and parasite challenges, feed efficiency research plays a key role in improving biological performance under environmental constraints. Studies conducted with locally adapted breeds have demonstrated that resilience and efficiency traits may respond differently across ecological contexts, reinforcing the need for region-specific investigations (Rodrigues et al. 2024; Pires et al. 2021).

Furthermore, the increasing internationalization of research networks highlights the importance of genotype-by-environment interactions in shaping feed efficiency expression. Evidence suggests that findings obtained under temperate intensive production systems may not fully capture the biological and management challenges faced in tropical and grazing-based environments, where animals are exposed to greater climatic variability and nutritional constraints. Consequently, expanding collaborative research across diverse ecological contexts is essential to develop more globally relevant and adaptable efficiency strategies. This perspective reinforces the need for integrative studies that consider environmental heterogeneity when interpreting variation in residual feed intake and its implications for livestock sustainability (Cantalapiedra-Hijar et al. 2018; Martin et al. 2021).

### Journal structure and scientific maturity of the field

The concentration of publications within a core group of specialized journals is a typical characteristic of scientifically mature research fields. Reviews on feed efficiency and animal production systems have emphasized the role of established journals in promoting methodological standardization, facilitating scientific debate, and supporting cumulative knowledge development (Kenny et al. 2018; Marino et al. 2023).

At the same time, the dispersion of additional studies across journals from complementary disciplines reflects the growing interdisciplinary nature of feed efficiency research. The integration of perspectives from microbiology, physiology, genetics, and environmental sciences has been increasingly recognized as essential to understanding the multifactorial determinants of RFI variation (O’Hara et al. 2020; Liu et al. 2022).

### Scientific leadership and collaboration networks

Authorship patterns characterized by a combination of highly productive researchers and a broad base of contributors are consistent with those observed in consolidated scientific domains. Long-term engagement of leading research groups has been reported as a key factor driving advances in feed efficiency studies, particularly in areas such as genetic selection, metabolic efficiency, and rumen function (Cantalapiedra-Hijar et al. 2018; Kenny et al. 2018).

The extensive collaboration networks identified in this study reinforce the importance of interdisciplinary and international approaches to addressing the biological complexity of feed efficiency. Collaborative research has been fundamental for integrating datasets from different production systems (McManus et al. 2023) and for improving the robustness and applicability of efficiency indicators across environments (Martin et al. 2021).

### Implications for future research and livestock sustainability

The bibliometric patterns observed in this study indicate a growing integration of feed efficiency research with themes such as methane mitigation, rumen microbiome dynamics, and metabolic efficiency reflects a shift toward holistic and systems-based approaches in animal science. Studies have demonstrated that RFI is associated with differences in digestive processes, microbial activity, and energy utilization, reinforcing its relevance for sustainable livestock production (Velazco et al. 2017; Xie et al. 2022).

This evolution indicates that feed efficiency research is progressively moving from a purely performance-oriented perspective toward a framework that simultaneously considers economic viability and environmental sustainability. From a practical perspective, the increasing incorporation of feed efficiency traits into breeding programs highlights their potential to improve farm profitability and reduce production costs. Feed represents one of the largest expenses in ruminant systems, and selection for improved efficiency has been widely recommended as a strategy to enhance economic and environmental performance (Kenny et al. 2018; Ferreira et al. 2024). Consequently, the expansion of research on RFI is closely linked to the development of more economically efficient and competitive livestock production systems.

Advances in phenotyping technologies and precision feeding strategies have further contributed to the application of feed efficiency concepts in commercial production systems. Improved monitoring of feed intake, growth patterns, and metabolic responses enables more targeted management decisions and supports the development of resource-efficient production models (Martin et al. 2021). This shift reinforces the role of RFI not only as a selection criterion for genetic improvement but also as a tool to inform management practices and sustainability policies.

Furthermore, the increasing association between feed efficiency and environmental indicators, particularly enteric methane emissions and nutrient-use efficiency, highlights the relevance of RFI within global efforts to promote climate-smart livestock production (Martin et al. 2021). As livestock systems face increasing pressure to reduce their environmental footprint (Knapp et al. 2014; da Silva Soares et al. 2025), the integration of efficiency traits into breeding and management strategies is likely to play a central role in future production models. Nevertheless, significant knowledge gaps remain regarding the biological mechanisms underlying individual variation in feed efficiency and their interaction with environmental stressors, nutritional strategies, and production systems (Cantalapiedra-Hijar et al. 2018).

Addressing these challenges will require continued advances in high-throughput phenotyping, omics technologies, and long-term collaborative studies conducted across diverse environmental and management conditions. The international research networks identified in this analysis represent a critical foundation for advancing feed efficiency research toward more resilient and environmentally sustainable ruminant production systems.

### Emerging directions and strategic implications for future research

The bibliometric patterns identified in this study suggest a clear conceptual transition in feed efficiency research over the last decade. Earlier investigations focused primarily on residual feed intake as a zootechnical indicator based on intake–performance deviations, whereas more recent studies increasingly integrate high-throughput phenotyping tools, precision livestock farming approaches, and microbiome-based indicators. This evolution reflects a shift toward “smart phenotyping”, in which biological efficiency is evaluated through multidimensional datasets combining physiological, behavioral, and molecular information (Martin et al. 2021; Marino et al. 2023).

A particularly important research trend observed in the analyzed literature is the growing emphasis on the relationship between improved feed efficiency and enteric methane mitigation. Evidence indicates that animals with lower residual feed intake may present reduced emission intensity and differences in digestive efficiency and ruminal metabolism, reinforcing the relevance of efficiency traits within climate-smart livestock production strategies (Velazco et al. 2017; Batalha et al. 2020; da Silva Soares et al. 2025). These findings highlight the need for integrative research combining genetics, nutrition, rumen microbiology, and environmental assessment to better understand the biological mechanisms underlying variation in feed efficiency (Cantalapiedra-Hijar et al. 2018; O’Hara et al. 2020).

From a practical perspective, the results of this 10-year bibliometric assessment suggest that breeding and nutrition specialists working in tropical production systems should prioritize strategies aimed at improving resilience, resource-use efficiency, and adaptation to climatic variability. Studies conducted under heat stress and variable feeding conditions demonstrate that the expression of feed efficiency traits is strongly influenced by environmental factors, emphasizing the importance of locally adapted genetic resources and context-specific management strategies (Pires et al. 2021; Matos et al. 2024; Rodrigues et al. 2024). The integration of genomic selection, precision feeding technologies, and collaborative research networks is therefore likely to play a key role in improving productivity while reducing environmental impacts in future livestock production systems (Kenny et al. 2018; Martin et al. 2021).

## Conclusion

This bibliometric analysis provides a comprehensive overview of the global scientific development of research on RFI and feed efficiency in ruminants between 2015 and 2024. The results demonstrate a sustained expansion of scientific production, accompanied by increasing international collaboration and the consolidation of a structured research field supported by core journals and established research groups.

The geographic distribution of publications highlights the prominent role of countries with major ruminant industries while reinforcing the global relevance of feed efficiency research across diverse production environments. The evolution of thematic research areas indicates a progressive shift from a predominantly zootechnical interpretation of RFI toward integrative approaches linking genetic improvement, nutritional efficiency, rumen microbiology, and environmental sustainability.

Overall, the findings position feed efficiency research as a key scientific pillar for the development of climate-resilient and resource-efficient ruminant production systems. Future advances are expected to be driven by the integration of high-throughput phenotyping, precision livestock farming technologies, omics approaches, and interdisciplinary collaboration across contrasting production contexts.

## Data Availability

The data that support this study will be shared upon reasonable request to the corresponding author.
